# Speciation of organosulfur compounds in carbonaceous chondrites

**DOI:** 10.1038/s41598-021-86576-6

**Published:** 2021-04-01

**Authors:** Alexander Zherebker, Yury Kostyukevich, Dmitry S. Volkov, Ratibor G. Chumakov, Lukas Friederici, Christopher P. Rüger, Alexey Kononikhin, Oleg Kharybin, Alexander Korochantsev, Ralf Zimmermann, Irina V. Perminova, Eugene Nikolaev

**Affiliations:** 1 Skolkovo Institute of Science and Technology, Skolkovo, Moscow region Russia 143025; 2 Department of Chemistry, Lomonosov Moscow State University, Moscow, Russia; 3 National Research Center “Kurchatov Institute”, Moscow, Russia 123182; 4 Joint Mass Spectrometry Centre, Chair of Analytical Chemistry, University of Rostock, 18059 Rostock, Germany; 5 V.L. Talrose Institute for Energy Problems of Chemical Physics, N.N. Semenov Federal Center of Chemical Physic, Russian Academy of Sciences, Moscow, Russia; 6 Vernadsky Institute of Geochemistry and Analytical Chemistry of Russian Academy of Sciences, Kosygina 19, Moscow, Russia 119334; 7 Joint Mass Spectrometry Centre of Helmholtz Zentrum München, 85764 Neuherberg, Germany

**Keywords:** Mass spectrometry, Analytical chemistry, Geochemistry, Meteoritics

## Abstract

Despite broad application of different analytical techniques for studies on organic matter of chondrite meteorites, information about composition and structure of individual compounds is still very limited due to extreme molecular diversity of extraterrestrial organic matter. Here we present the first application of isotopic exchange assisted Fourier transform ion cyclotron resonance mass spectrometry (FTICR MS) for analysis of alkali extractable fraction of insoluble organic matter (IOM) of the Murchison and Allende meteorites. This allowed us to determine the individual S-containing ions with different types of sulfur atoms in IOM. Thiols, thiophenes, sulfoxides, sulfonyls and sulfonates were identified in both samples but with different proportions, which contribution corroborated with the hydrothermal and thermal history of the meteorites. The results were supported by XPS and thermogravimetric analysis coupled to FTICR MS. The latter was applied for the first time for analysis of chondritic IOM. To emphasize the peculiar extraterrestrial origin of IOM we have compared it with coal kerogen, which is characterized by the comparable complexity of molecular composition but its aromatic nature and low oxygen content can be ascribed almost exclusively to degradation of biomacromolecules.

## Introduction

Meteorites provide an important data source about the prebiotic chemistry. The systematic research on meteorite organic matter (OM) was started in the 1960-ies^1, 2^. Then, the fall of Murchison meteorite, which belongs to carbonaceous chondrites categorized as CM2, in 1969 provided a large amount of sample (about 100 kg) in a relatively good state of preservation. Historically, geochemical and cosmochemical studies split meteorite OM into two parts: soluble organic matter (SOM) and insoluble organic matter (IOM)^3^. The former refers to OM, which can be extracted by common organic solvents, such as CH_3_OH, C_2_H_5_OH, C_6_H_6_, C_6_H_5_OH, CH_3_Cl, CH_2_Cl_2_, CS_2_, whereas the latter refers to residual (insoluble) OM. A lot of studies have reported the presence of low molecular weight organic compounds in SOM^4^. These molecules might serve as building blocks for the synthesis of much more complicated structures by means of the Fischer–Tropsch process^5^. Various types of organic compounds were identified in SOM of carbonaceous chondrites using the sequential solvent extractions and different analytical techniques: aliphatic compounds^6^, aromatic compounds^7^, amino acids^5^, sugars^8^.

The major contribution to understanding molecular composition of meteorite OM was made by mass-spectrometry, which has always been a valuable tool for the geochemical applications^9^. The introduction by Fenn of the mild electrospray ionization method (ESI) made feasible analysis of various high-mass organic molecules^10^. Combination of ESI with high-resolution mass-spectrometry is a new chapter in the history of the extraterrestrial matter research^11^. Fourier transform ion cyclotron resonance mass spectrometry (FTICR MS) provides the highest resolving power and mass accuracy among the mass spectrometric techniques. This method allows for direct measurements of individual molecules in extremely complex mixtures, which laid grounds for “non-targeted” analytics^12^. The particular feature of non-targeted analysis is exploration of the entire molecular ensemble instead of a search for the known individual compounds. Application of FTICR MS led to identification of thousands organic components in SOM of Murchison^13^, Sołtmany^14^ and Chelyabinsk^15^. However, the molecular composition of IOM, which comprises the largest part of total meteorite OM—up to 70%^16^—is not revealed yet by high-resolution mass spectrometry. The study of IOM is important in the context of understanding the Solar system evolution and processes taking place in the parent asteroid body^17^, e.g. alteration of organic matter by heat^18^ and/or water^19^.

The significant lagging of IOM behind SOM studies can be partially explained by both technical and ideological issues. First of all, extraction of IOM is literally the destruction of meteorite. The general approach, which has been implemented to IOM isolation in classical geochemistry, included SOM removal by a sequential extraction with organic solvents followed by destruction of carbonates and silicates using HCl and HF treatment, respectively^20^. This procedure is harsh and it was shown that impact of acids results in both loss of organic carbon^21^ and transformation of IOM macromolecules^22^. In addition, analytical methods, which have been applied in IOM studies, had some inherent limitations. For example, pyrolysis revealed a number of sub-structures in the IOM matrix, however, it is impossible to distinct between the real parts of IOM and artifacts formed due to secondary reactions. At the same time, application of laser desorption from the IOM surface resulted in detection of limited number of small molecules. Aromatic molecules like naphthalene or anthracene, and other PAHs were found in Allende meteorite^23^.

Demineralized residue can be used for a non-destructive investigation by solid-state ^1^H and ^13^C NMR spectroscopy^24, 25^. It was suggested that Murchison IOM is composed primarily of highly substituted single ring aromatics, substituted furan/pyran moieties, and highly branched oxygenated aliphatic groups. At the same time, NMR provides only integer characteristics of the carbon distribution without information on individual molecules. This shows a lack of widely accepted strategy in the analysis of chemical structures of IOM. The structural models of IOM were proposed on the basis of different analytical data (solid-state NMR, Fourier transform infrared spectroscopy (FTIR), electron spin resonance spectroscopy (ESR), X-ray absorption near-edge spectroscopy (XANES), high-resolution electron microscopy, thermal and chemical degradations)^26^. In many studies IOM was compared to low-rank coal due to its extreme molecular and structural heterogeneity^27^. However, the applied methods conveyed rather information about functional groups or structural fragments of the molecules than on the molecular ensemble of IOM.

In this work, we focused on the analysis of alkali soluble IOM fraction of Murchison and Allende meteorites using FTICR MS. In order to overcome the tolerance of FTICR MS to structural isomers, we applied its combination with H/D isotopic technique, which is widely used to study various complex mixtures^28^.

## Results

### XPS characterization of IOM samples and alkaline-extracts

**Figure 1:**
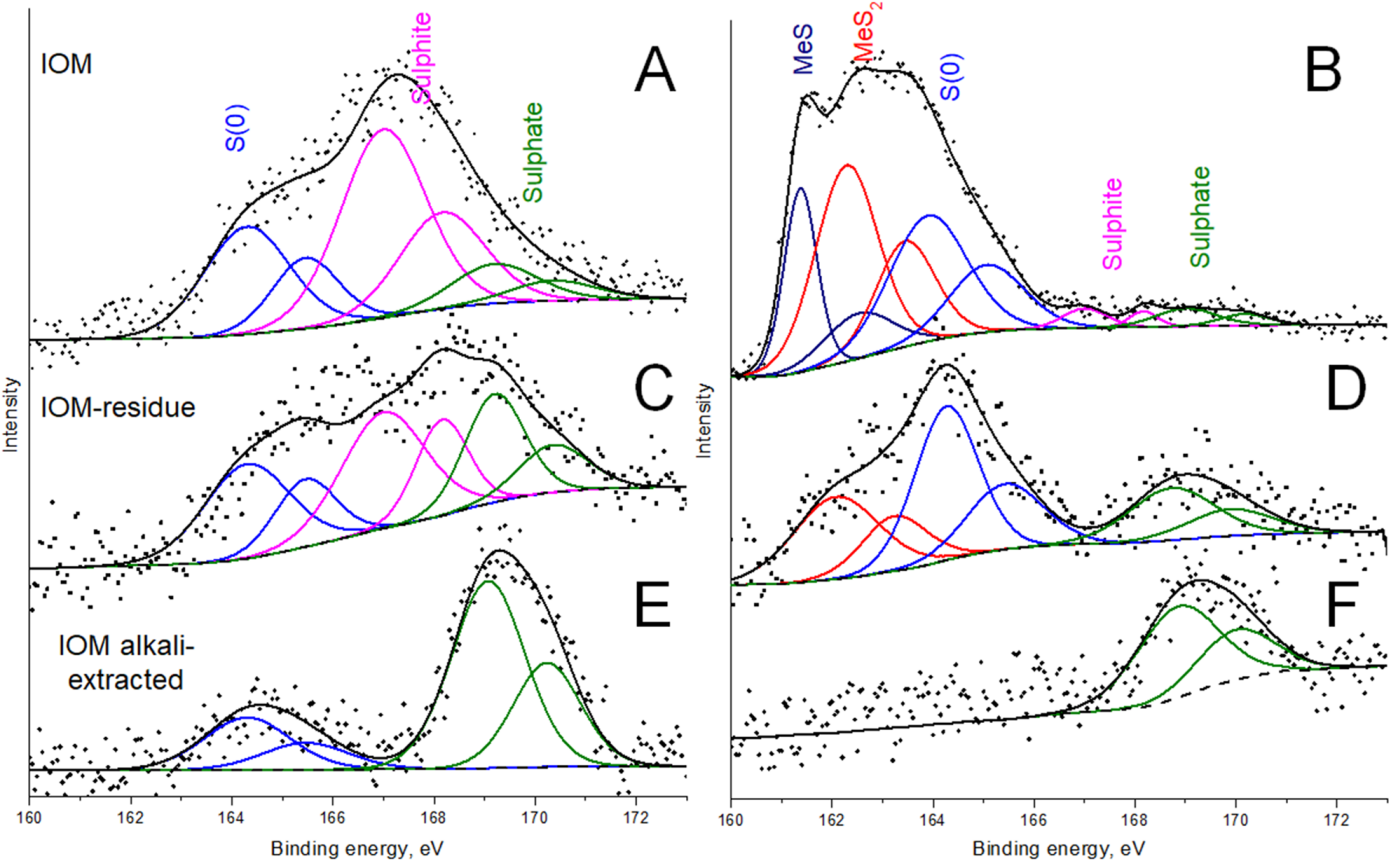
XPS spectra of the IOM fractions. Left and right panels show results for Murchison and Allende, respectively.

**Figure 2:**
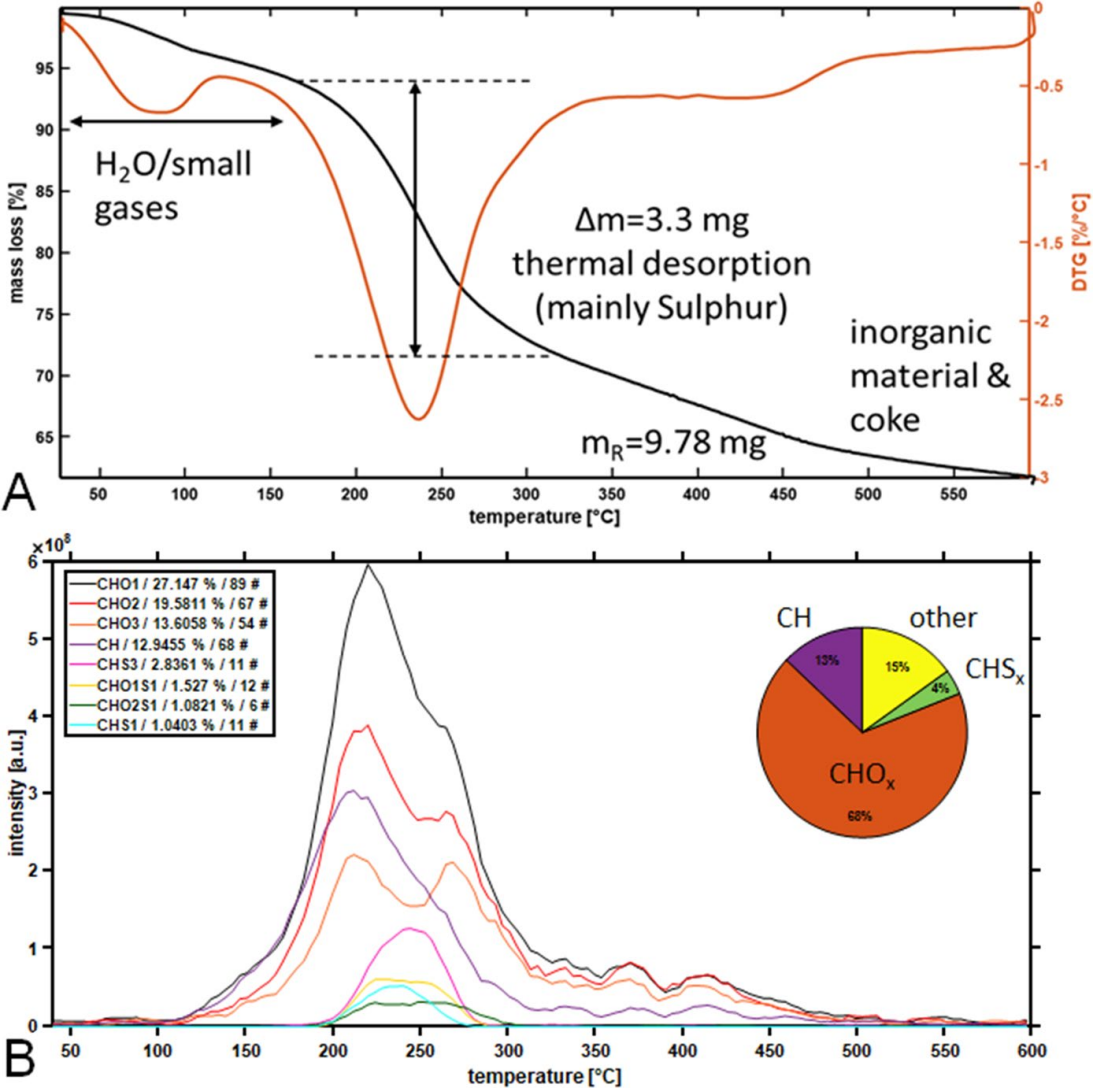
(**A**) Temperature-resolved mass loss and differential mass loss curves normalized to the 15 mg input weight Murchison IOM. (**B**) Temperature-response of the individual compound classes of Murchison IOM organic footprint. The inset reflects the proportions of the three dominant classes pure hydrocarbons (13%, CH), oxygenated organics (68%, CHO_x_) and Sulphur-species (4%, CHS_x_). Other classes, mainly oxygenated Sulphur-species, are summed up and given as rest with 15%.

After demineralization of parent materials, the weights of 2.6 mg and 1.3 mg of IOM were obtained in case of Murchison and Allende meteorites, respectively. Alkaline extraction yielded: 0.4 mg (31% of IOM) and 0.15 mg (23% of IOM) for Murchison and Allende, respectively. For comparison of alkaline extracts, XPS spectra for all fractions were obtained. XPS enables to analyze the chemistry of heteroatoms on material surface, similar to XANES, which has been previously applied to compare Murchison and Allende IOM. The deconvoluted spectra of the sulfur line S2p for the samples used in this study are presented in Fig. 1. The Allende IOM (Fig. 2B) consists of a set of S2p 3/2 and 1/2 lines corresponding to the sulfides and disulfides (MeS^-2^, MeS_2_), elemental sulfur S^0^ and low band of sulphite^+4^ and sulphate^+6^, while Murchison IOM was enriched with oxidized sulfur bands. This was in agreement with the previous XANES examination of Allende IOM^29^. Inorganic sulfur is usually well resolved, however, differentiation between inorganic and organic sulfur even with similar valence state is not straightforward in case of XPS analysis. Taking into account that the IOM was isolated by the harsh acidic treatment (HCl and HF) which should have removed a major part of inorganic salts, we refer the observed bands to organosulfur compounds. The band in the region of reduced sulfur (thiols) is harder to interpret since pyrrhotite, pyrite and other sulfides residues are often observed in IOM even after harsh acidic treatment. In case of Murchison IOM (Fig. 1A), we observed a lack of MeS and MeS_2_ bands and dominance of sulphite- and sulfate-like sulfur atoms. In terms of organosulfur compounds this might be indicative of high abundance of sulfonyls and sulfonates, which were previously reported for the Murchison IOM^30^. Alkali-extracted fractions from IOM samples and the solid residues showed the similar trends: in both cases alkaline extraction led to enrichment of oxidized sulfur state, while in case of solid residue the distributions shifted toward more reduced sulfur states (Fig. 1C–F). The difference between the two IOM materials used in this study retained also in case of the residues (Fig. 1C,D). At the same time, the alkali isolate from Murchison was enriched with the less oxidized sulfur atoms as compared to the Allende (Fig. 1E,F).

### Thermal analysis of Murchison IOM hyphenated to FTICR MS

The thermogravimetric analysis (TGA) of the Murchison IOM was characterized by a typical water loss and emission of small molecules and gases (not detected with the mass spectrometric hyphenation) at 60–120 °C with a loss of roughly 6% of the sample weight (Fig. 2A). A dominant desorption step at 170–300 °C accounts for a loss of roughly 22% (3.3 mg). In the pyrolysis region (> 400 °C) only a less dominant step at 420–460 °C with a mass loss of ~ 7% was found. Roughly 10 mg (62%) of the sample weight remained after pyrolysis as inorganic material or coke in the crucible.

The mass spectrometric response of the abundant desorption step is dominated by the decomposition of elemental sulphur. The decomposition products were detected as various sulphur-cluster (S_n_) with the S_8_ as the most stable species. The ring containing structures were found with the highest abundance (Fig. S1). The signals can be easily identified based on the high mass defect and characteristic isotopic pattern. Species from S_5_ (*m/z* 159) up to S_9_ (*m/z* 287) were found. The similar Sulphur decomposition pattern was reported for elemental Sulphur with mass spectrometry coupled to TGA^31^. In addition to this inorganic pattern, the footprint of organic compounds was observed between *m/z* 150 -450 (inset Fig. S1). The found signals belong mainly to pure hydrocarbons (CH-class, 13%), oxygenated organics (68%, CHO_x_) and Sulphur-species (4%, CHS_x_). Other classes, mainly oxygenated Sulphur-species accounted for 15% (Fig. 2B). These species were not present in the blank measurements, and they clearly exposed a temperature trend. The species were emitted during the dominant desorption step and peaked between 200 and 280 °C. Figure 2B illustrates this temporal behavior for the eight most abundant classes.

**Figure 3:**
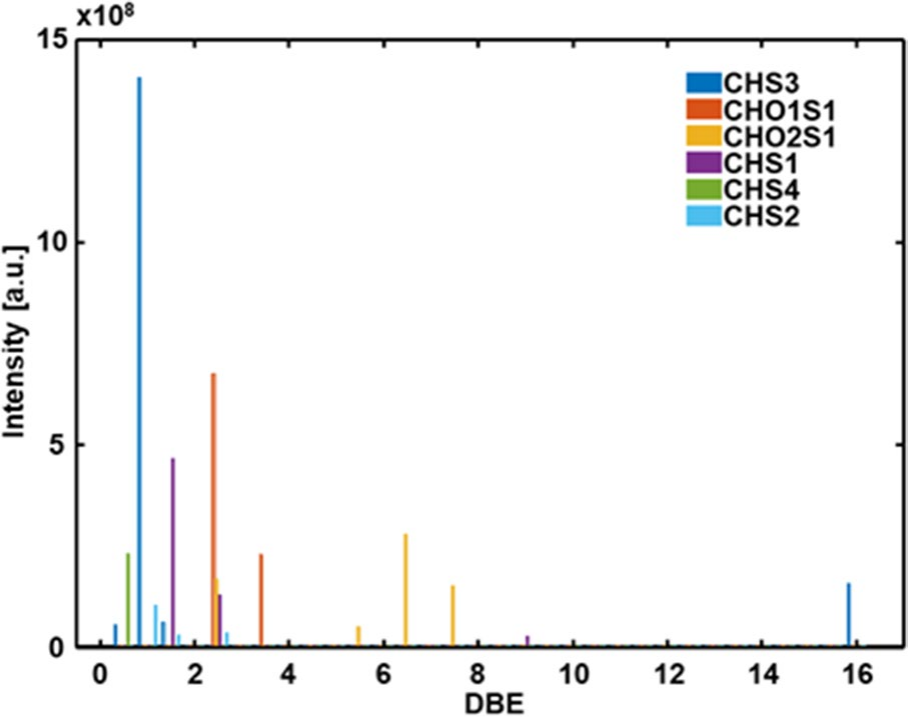
Summed double bond equivalent (DBE) distribution for the Sulphur-containing compound classes in Murchison IOM. The oxygenated Sulphur-species are found to have rather low DBE for one Oxygen-atom (CHSO_1_) and increased aromaticity for the species with two Oxygen-atoms (CHS_1_O_2_).

Along the clearly dominating oxygenated classes (CHO_x_), the oxidized organosulfur compounds were also found with a total abundance of about 2.5% (CHS_1_O_1-2_). The aromaticity of those signals can be accessed utilizing the double bond equivalent (DBE) (Fig. 3). Organosulfur compounds with one Oxygen-atom (CHS_1_O_1_) were found to have low DBE value (DBE 3) corresponding to thiophenic structure, whereas the compounds with two Oxygen-atoms (CHS_1_O_2_) had the higher unsaturation (DBE 7), which might be indicative of benzothiophenic structures. Also, S-containing compounds (both oxygenated and pure CHS) with DBE < 3 were observed, which might indicate the presence of thiols in the Murchison IOM sample.

### FTICR MS analysis of the alkali isolates from the Allende and Murchison IOMs

**Figure 4:**
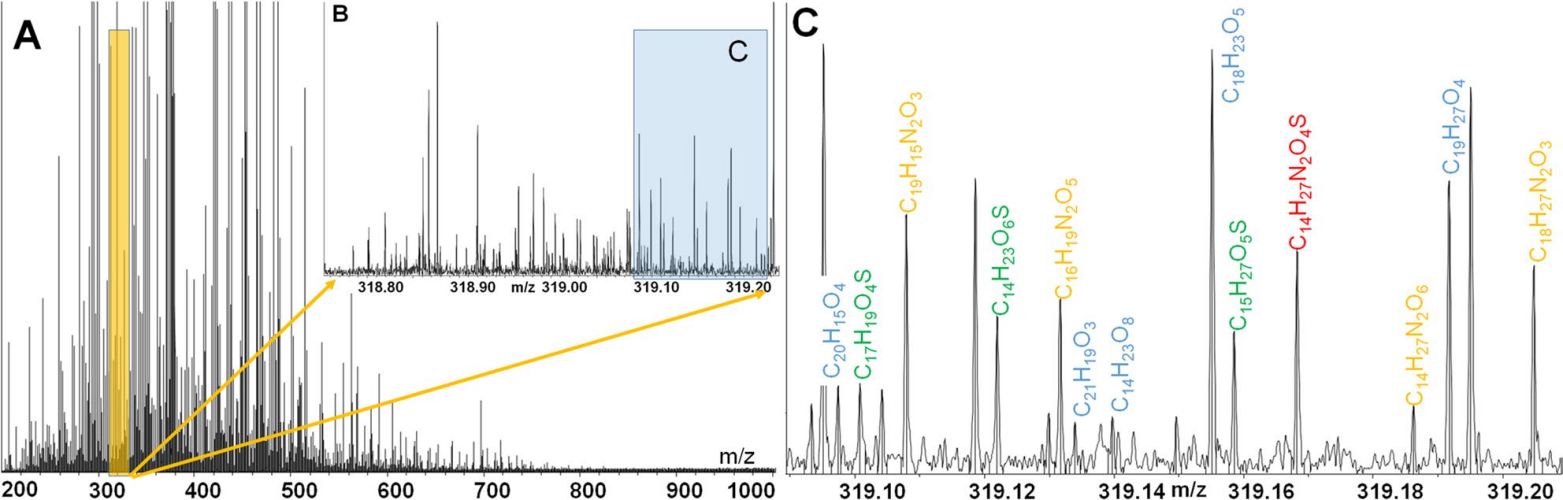
(**A**) FTICR mass-spectrum of alkali-extracted IOM (on the example of Murchison), (**B**, **C**) Insets show magnified fragments within 0.4 and 0.1 Da, respectively.

The negative ion ESI FTICR mass-spectrum of the alkali extracts from the Murchison and Allende IOM contained 21,648 and 21,161 well-resolved peaks, respectively, with signal-to-noise ratio exceeding 4. All peaks within mass range of 200–1000 m*/z* were singly charged ions (Fig. 4A). The mass spectra of alkali-extracted IOM were characterized with extreme molecular diversity similar to SOM^13^ resulting in the presence of 87 different mass-signals on a single nominal mass (Fig. 4B). More than 10 elemental compositions were assigned within 10 mDa mass window for a nominal *m/z* of 319 (Fig. 4C). In spite of a use of PPL cartridge, ESI(−) spectrum contained a significant amount of salt adducts including Fe with a mass of 55.934 amu, which was identified based on mass defect values of about 0.5 Da. The presence of metal–organic compounds has been previously shown for 61 meteorites of different petrological types^32^.

**Figure 5:**
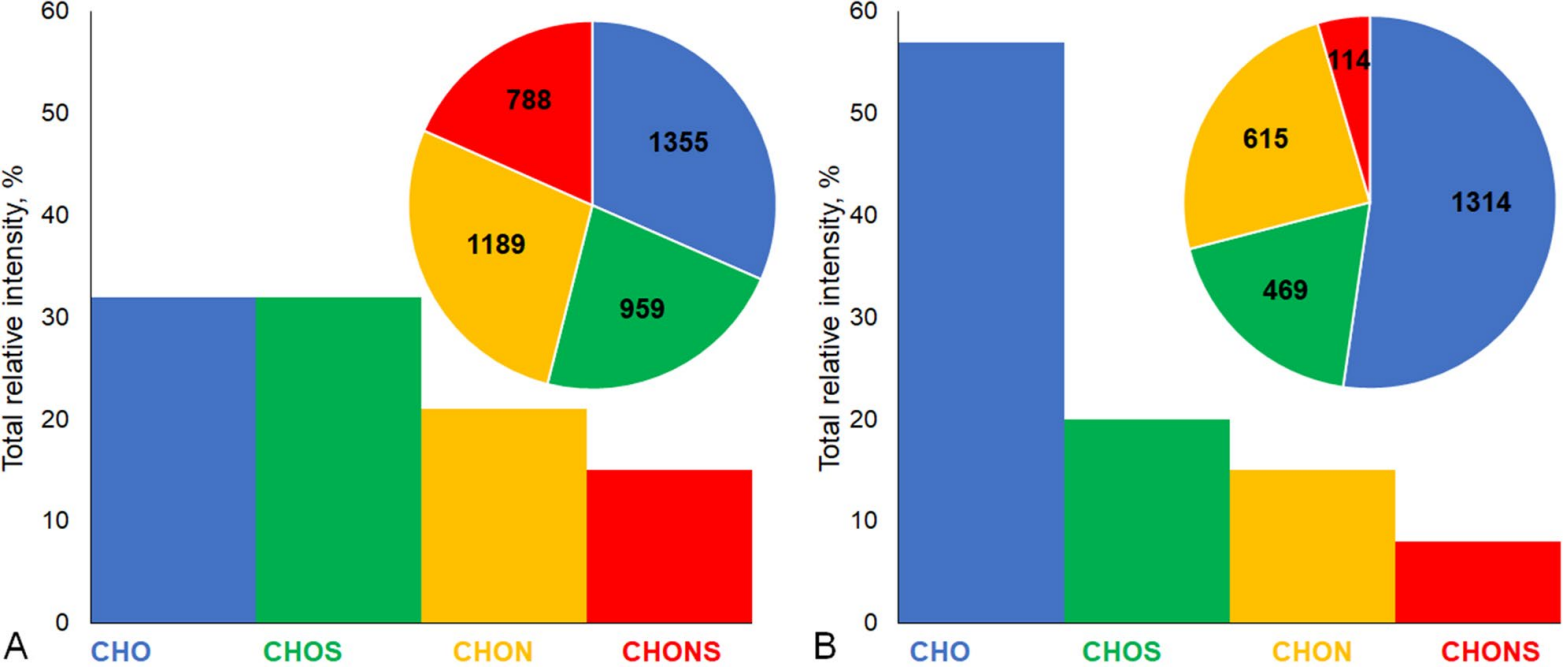
Distribution of different molecular compositions determined from the FTICR MS data assignments: (**A**, **B)**—summarized relative intensity and a number of formulae for alkali-extracted IOM from Murchison and Allende, respectively.

High resolution FTICR MS enabled identification of 4291 and 2512 formulae in the Murchison and Allende isolates, from which 1545 were common for the both samples (Fig. 5). The lower amount of formulae in the Allende IOM is in agreement with its higher petrological type and deeper thermal metamorphism as compared to Murchison. The similar trend has been described for methanol-extracted SOM of Murchison, Allende and other meteorites^32^. Unlike the reported FTICR MS results on the Murchison SOM^12^, in the both alkali extracts CHO molecular compositions were most abundant. Additionally, CHOS molecular compositions were highly abundant, especially, in case of Murchison reaching 30% of a total intensity. The similar results were reported for the methanol extract of L6 Soltmany meteorite, which underwent significant thermal alteration^14^. According to FTICR MS data, the average molecular formulae of the alkali-extracted IOM from the Murchison and Allende, were C_47_H_65_O_13_N_1_S_1_ (O/C_n_ = 0.28 and H/C_n_ = 1.38) and C_76_H_117_O_21_N_1_S_1_ (O/C_n_ = 0.28 and H/C_n_ = 1.54). Both samples are more oxygenated and substantially more saturated as compared to reported bulk IOM empirical formulae^29^. For example, for Murchison IOM the combustion technique showed H/C = 0.72^33^ and even 0.17 for Allende IOM^17^. This might be indicative of underestimation of aromatic units by FTICR MS.

**Figure 6:**
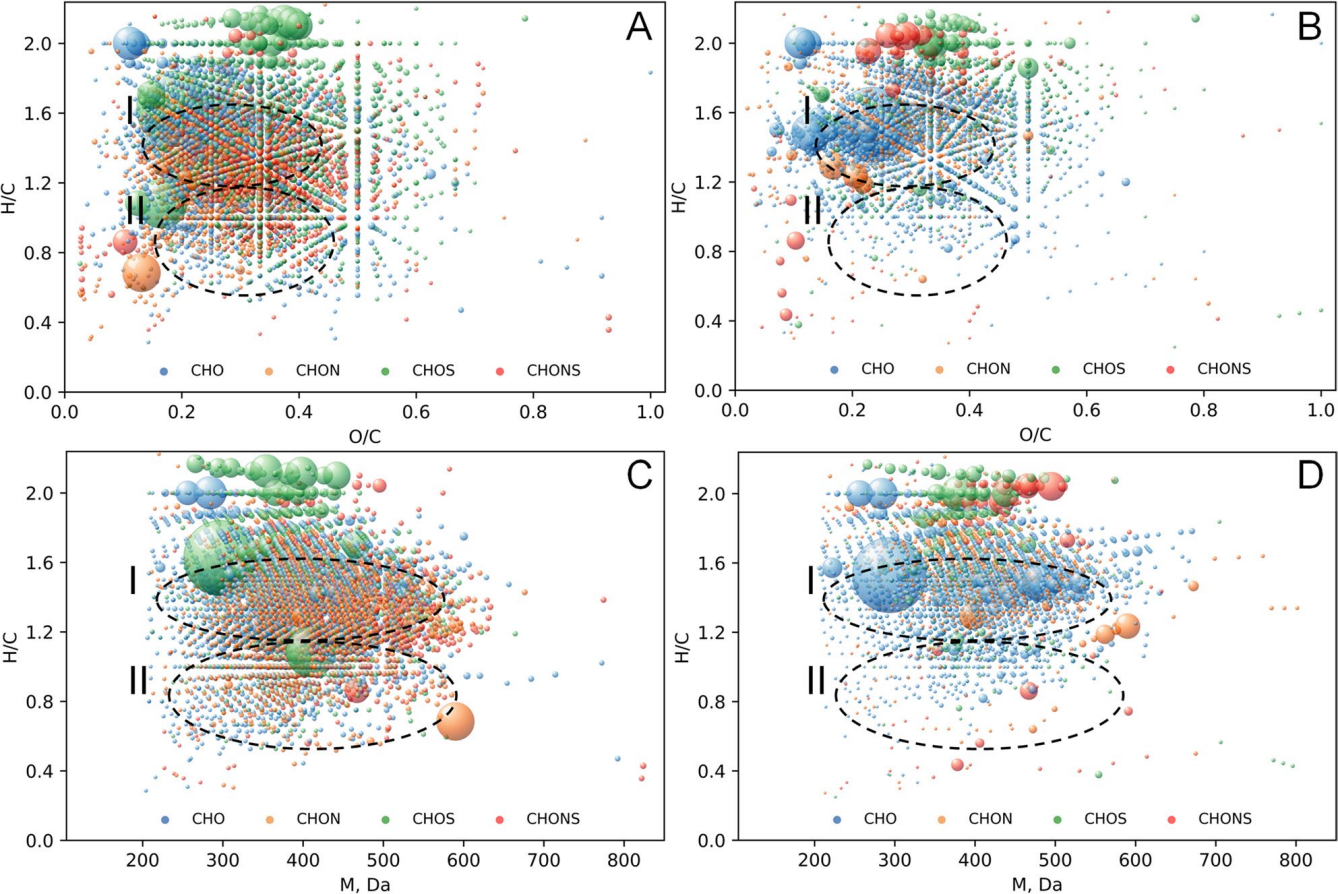
Van Krevelen diagrams (**A**, **B**) and H/C versus molecular mass diagrams (**C**, **D**) for molecular compositions identified in the alkali-extracted IOM samples. The dot size represents relative intensity of the corresponding peak in the FTICR mass spectrum. CHO, CHON, CHOS, and CHONS compositions are highlighted in blue, orange, green, and red, respectively. Regions I and II correspond to substituted and condensed aromatic species. Left and right panels correspond to Murchison and Allende extracts, respectively.

The conventional approach to FTICR MS data visualization is van Krevelen diagram (H/C vs O/C plot)^34^. It can be also extended by H/C versus molecular mass (M) diagram. Figure 6 shows the resulting diagrams for CHO, CHOS, CHON and CHONS molecular compositions. It was previously shown that the Murchison SOM was mostly comprised of the low-oxidized saturated compounds with O/C < 0.2^13^. However, water-extracted fraction of Murchison was populated by aromatic compounds with alkyl substituents^35^. Alkali may dissolve lesser polar compounds. Accordingly, the alkali extracted IOMs (Fig. 6A-B) were characterized by the dominant contribution of moderately oxidized unsaturated compounds with H/C < 1.6. The both samples were compared to the similar extract from lignite, which is mainly represented by aromatic and condensed aromatic species^36^. It should be noted that the lignite IOM was considered for a long time as a terrestrial model for the carbonaceous chondrites^26^. The results of FTICR MS analysis of the coal alkali extracted IOM are presented in Fig. S2 as van Krevelen diagrams. It can be seen that the coal OM is characterized by much higher contribution of aromatic compounds than the alkali-extracted IOM from the both carbonaceous chondrites used in this study. The average molecular formula of the coal OM extract is C_92_H_94_O_36_N_1_S_0.2_, which has the lesser saturation degree as compared to the extraterrestrial IOM (H/C ratio 1 vs 1.38 or 1.54). Unlike the IOM, the alkali-extracted coal OM is almost depleted with sulfur, and the identified CHON and CHOS compositions are depleted with the oxidized species: this is the major difference between the compared samples. The comparison of Murchison IOM to coal OM is justified by highlighting the conventional classes of organic compounds in van Krevelen diagrams^37^. Biomacromolecular residues like lignin and condensed aromatics contribute the most to the alkali-extracted fraction of coal as it is shown in Fig. S2^36^. Obviously, no biomolecule residues are to be searched in extraterrestrial materials. Still, in terms of molecular composition the same regions are highly populated in case of Murchison IOM and coal OM. Based on chemical constraints to atomic compositions, the determined Murchison alkali-extracted IOM components can be conservatively assigned to aromatic molecules with functionalized aliphatic substituents (region I) and condensed aromatic compounds (region II)^37^. This corroborates well the NMR data on the demineralized Murchison IOM witnessing its substantial aromaticity along with abundant aliphatic substituents^24^. The comparison of Murchison to Allende shows the depletion of the latter with condensed aromatic structures. Still, H/C versus M diagrams (Fig. 6C,D) highlight similarity in molecular composition of the both IOM samples: the molecular masses increased along with a decrease in H/C ratio values in high H/C region (> H/C ~ 1.2), and reverse trend was observed within the low H/C values (< H/C ~ 1.2). The trend observed in our study might reflect condensation patterns of the organic constituents of IOM. The similar process is observed for lignite in Fig. S2(D-F) brought about by oxidative coupling of their phenolic precursors^38^.

### Determination of oxidation pattern of IOM components

**Figure 7:**
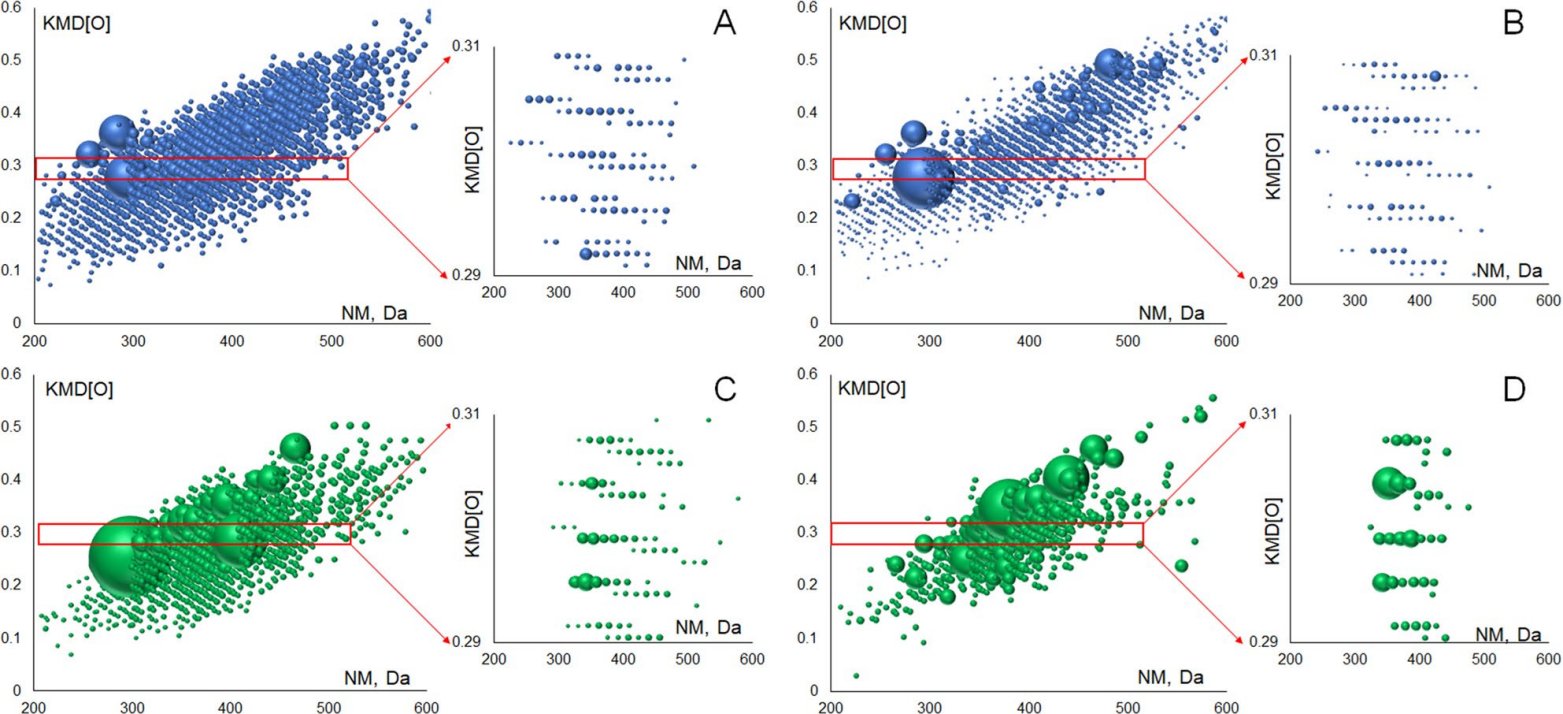
The Kendrick diagrams (O-based) for CHO (**A**, **B**) and CHOS (**C**, **D**) molecular compositions. The insets show the magnified diagram fragments with several homologues series. Left and right panels correspond to alkali-extracted IOM from Murchison and Allende, respectively.

Connection between CHO and CHOS compounds was evaluated by plotting oxygen-based Kendrick mass defect (KMD[O]) diagrams for the CHO and CHOS compounds (Fig. 7). The obtained diagrams show that in both cases CHO and CHOS compounds contain molecules connected by oxidation series. Therefore, oxygen enrichment could not be explained exclusively by reactions of organic compounds with sulfates from inorganic matrices. Moreover, it was previously suggested that the Murchison OM was oxidized during hydrothermal alteration in the parent body^24, 39^. The results in Fig. 7 might indicate occurrence of extensive oxidation during both CHO and CHOS species formation. The insets in Fig. 7 demonstrate that both carbon skeleton and organic sulfur could undergo oxidation. This contradicts to the dominant role of sulphates during formation of CHOS compounds in alkali-extracted IOM as it was suggested for SOM^14^. Yet, it should be noted that in case of Allende, the oxidative series are significantly shorter for CHOS as compared to CHO molecular compositions, or both types of compositions, in case of Murchison.

### Application of H/D exchange for enumeration of mobile protons in S-containing organic compounds in the alkali isolates of Murchison and Allende IOM

**Figure 8:**
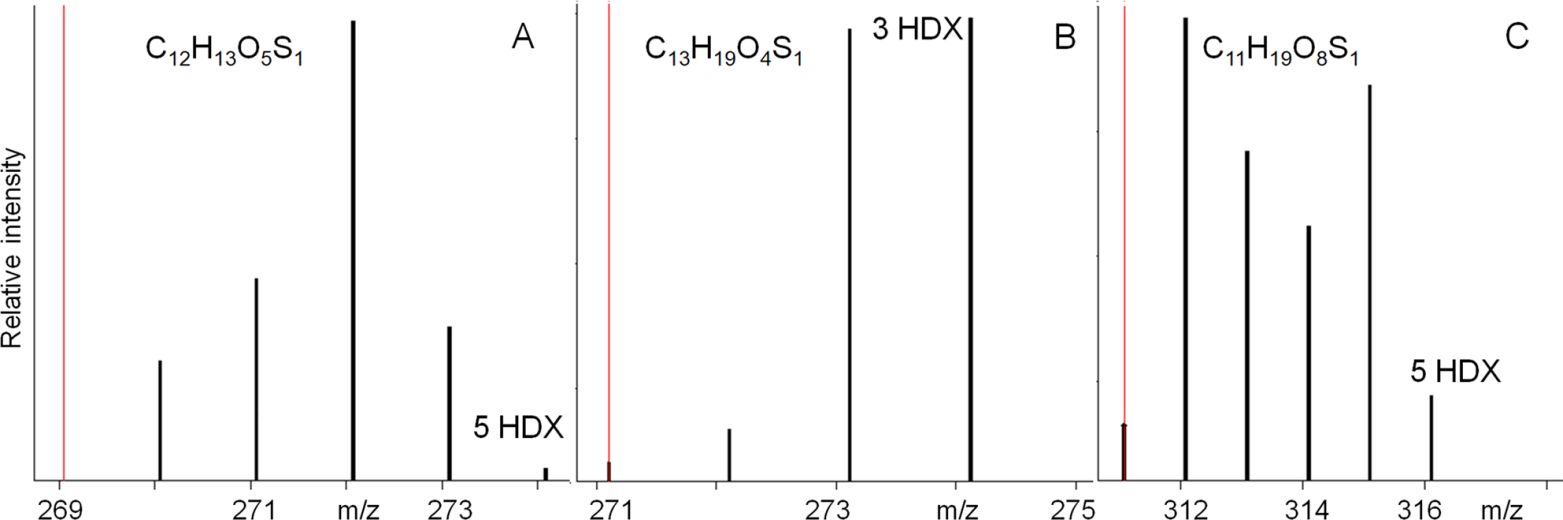
HDX series extracted from the full FTICR mass-spectrum of the labeled Murchison IOM sample. The red line designates *m/z* value of the parent-ion.

In order to specify compound classes, which contribute into CHOS compositions of the both IOM alkali extracts, we applied H/D exchange (HDX). Figure 8 shows reconstituted mass-spectra fragment corresponding to HDX series of the several parent-ions in the native sample. Due to ultra-high resolution power FTICR MS allows for an unambiguous identification of HDX series. The length of exchange series equals to the number of mobile protons in the molecule minus one H-atom responsible for ionization in ESI (−). This is because C–H bonds remain intact during HDX without acid/base catalysis^40^. For example, we observed 5 HDX for C_12_H_13_O_5_S_1_ molecular formula, which was identified in the alkali-extracted IOM of Murchison. This indicates that six protons (HDX + 1) were bound to heteroatoms in the compound from IOM with matching molecular composition.

**Figure 9:**
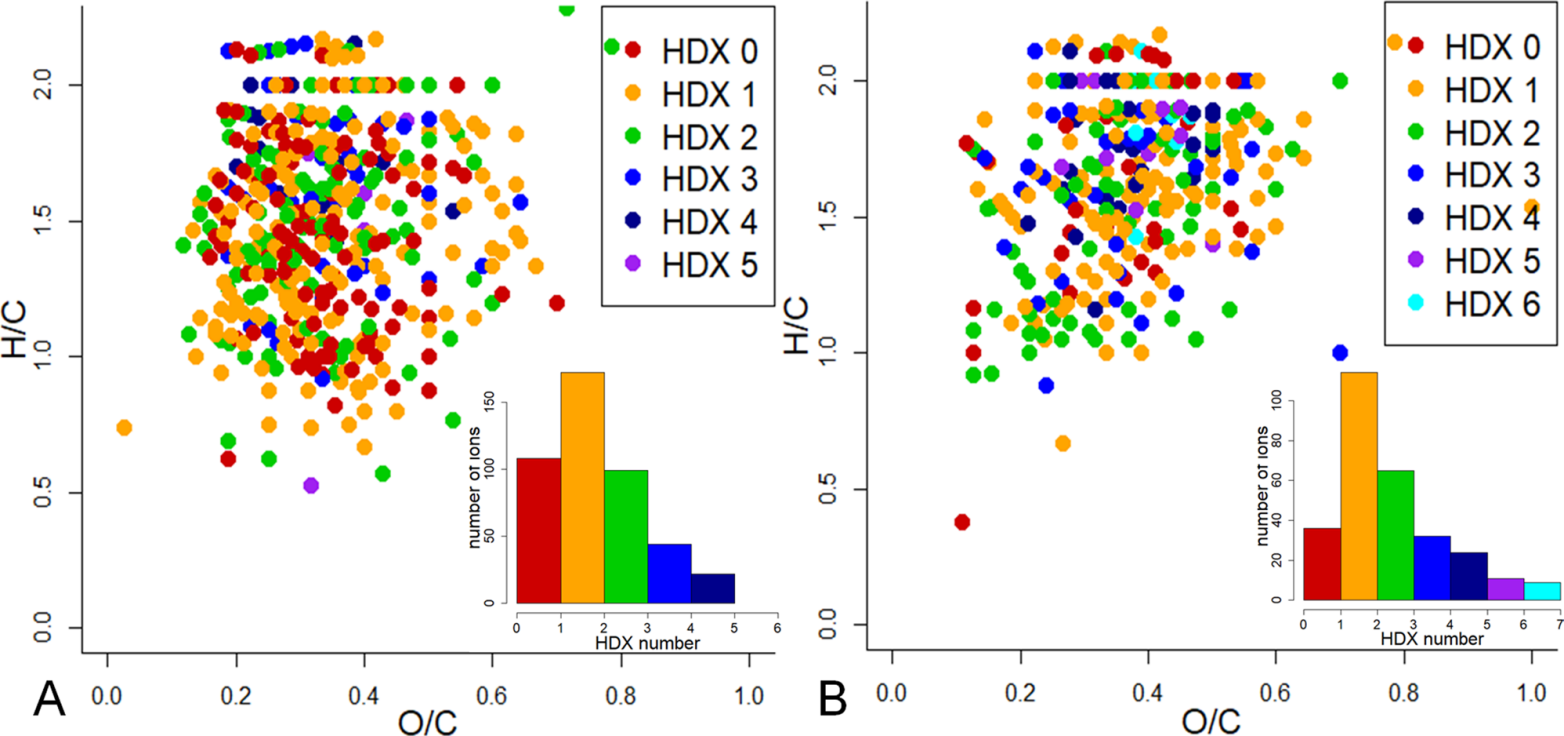
The van Krevelen diagram plotted for 445 and 291 CHOS assignments from the mass-spectra of Murchison (**A**) and Allende (**B**) IOM samples, respectively. The color coding scheme is used for a number of mobile protons. Insets show distribution of ion number with different amount of exchanges.

The HDX series were robustly calculated for the 445 and 291 CHOS formulae identified from the mass-spectra of the native Murchison and Allende IOM samples, respectively. The obtained results were plotted in van Krevelen diagram using color coding for the number of HDX identified for each assigned ion. From Fig. 9 it can be deduced that the distribution of HDX numbers is similar for both samples and varied from 0 to 5 (in case of Allende, also 2 ions with 6 HDX were found). In both cases, the ions with 1 HDX were the most abundant. It should be noted that the highly oxidized species with O/C > 0.5 were depleted with mobile protons as compared to the compartments with O/C ≈ 0.5. This could be explained by structural differences of the corresponding compounds. Indeed, the KMD[O] statistics (Fig. 7) suggests that the higher oxidized compounds with the longer O-homologue series contain SO_3_H-groups (the valence state of sulfur is + 6). At the same time the less oxidized saturated compounds may preferably contain thiolic groups (the valence state of sulfur is − 2) and alcoholic groups: all of them undergo HDX.

## Discussion

### Evaluation of the pristine character of the extracted IOM

In this research we suggested a novel approach to IOM studies by solubilizing it in aqueous alkali. Taking into account that impact of both strong acids (HCl, HF) and alkali (NaOH) may alter the nature of pristine IOM^21^, it was necessary to justify the relevance of the demineralized and alkali isolates to the parent material. The yield of extraction was relatively high − 31% and 23% for Murchison and Allende, respectively. For evaluation of material pristine character, we performed XPS analyses of the parent IOM, of the alkaline isolates, and the solid residues after extraction of the both meteorites used in this study. Inspection of Fig. 1 shows that the alkali isolates and residues obtained from the both samples contain the species with the same sulfur valence state as the parent samples, which might be indicative that they are the parts thereof. The only exception is the MeS state of sulfur in Allende, which was observed only in the parent IOM sample. This can be attributed to the hydrolysis of inorganic sulfides during extraction. It should be noted that XPS is only surface layer-sensitive. That is why an absence of the signal could be erroneously interpreted as an absence of the corresponding atom types in the sample: they may present in the matrix, but be out of reach of the X-ray beam. This might explain why the reported study on sulfur-speciation in Murchison measured by high-energy XANES^30^ detected full range of sulfur-types, while we could not observe thiols both in the IOM and isolates thereof. In case of the Allende meteorite, the IOM sample was depleted with oxygenated sulfur—the intensity of the corresponding bands were low, which corroborates well the results of the previous investigation of Allende using XANES^29^. At the same time, the alkaline extract was enriched with the oxygenated sulfur, which can be connected with the extraction procedure. In the case of the Murchison meteorite, the alkaline extract was also enriched with oxygenated sulfur, but it also contained more reduced sulfur and atomic sulfur bands (Fig. 1E). This finding agrees well with the results of the orthogonal thermal analysis of the evolved gases using FTICR MS: it revealed abundant S^0^ clusters in the mass-spectrum of the parent IOM.

Thermal analysis of Murchison IOM hyphenated to FTICR MS also provided a support for the only negligible reactions to form S-bearing organic compounds during demineralization of IOM by conventional but harsh acidic treatment. In fact, acidic hydrolysis results in the release of the hydrogen sulfide, which may further react with unsaturated C_x_H_y_ and oxygen containing species yielding formation of new S-bearing organic compounds^9^. That consideration is particularly important for molecular examination of IOM and its extracts, and possible by-products of the laboratory process should be unambiguously distinguished from the indigenous molecules. In Fig. S3 the cumulative abundances of species with different DBE values are plotted. For the CH- and CHO_1-2_– classes of Murchison IOM we observed wide distributions of intensity (Fig. S3A-C) from non-aromatic structures with DBE < 4 to the highly unsaturated likely aromatic compounds with DBE > 8. The presence of diverse non-aromatic and condensed compounds has been recently shown by application of laser desorption ionization of chondrite IOM coupled to FTICR MS^42^. The DBE-distributions of CHS and CHO_1-2_S compounds in Murchison IOM (Fig. S3D-F) were substantially different. CHS compounds were represented by compounds with DBE = 2 and 3, exclusively. Based on DBE values these species may be assigned to thiols and thiophenes, in which thiols possessed higher abundance. While in case of CHO_0,1_–species the maximum of abundance was attributed to compounds with high DBE. In case of reaction on the organic matrix with the released hydrogen sulfide we would observe at least low-abundant highly unsaturated CHS species. Additionally, CHO_2,3_- and CHO_2_S– species also represented different molecular profiles. Together these observations provided by thermal analysis indicate the S-bearing species were not formed significantly by the IOM isolation.

### Uniqueness of the molecular composition of the alkali extracted IOM as compared to the terrestrial model one

Despite seeming similarity of the molecular space of the IOM samples and the bulk distribution of molecular species of the coal IOM used in this study, the deeper consideration showed significant differences. The coal IOM was characterized by the dominant contribution of the CHO molecular series mostly attributed to aromatics. These aromatic moieties have distinctively biomacromolecular origin. Biological degradation led to oxidation of aliphatic fragments to CO_2_ and to accumulation of aromatic compounds. Otherwise, the chondrites IOM is characterized by the dominance of N,S-containing compounds, which corroborates well a decrease in H/C ratio and accumulation of aromatic heterocycles during hydrothermal alteration of IOM^41^. Therefore, very different synthetic pathways for the chondrite IOM and coal IOM could not result in producing closely related structures. This similarity is simply a result of a projection of numerous isomers onto van Krevelen diagram.

Additionally, homologue distribution of both CHO and CHOS constituents in the coal sample also highlights peculiar features of the chondrite IOM. The KMD[O] diagrams plotted for the species identified in the coal IOM isolated by the same procedure (Fig. S4(A)) revealed the presence of extensive O-homologues in CHO species. However, unlike isolates from chondrites the CHOS compounds do not reveal the presence of oxygen series in the case of coal OM (Fig. S4B). This can be explained by a lack of selectivity in the case of IOM transformation, in which both CHO and CHOS molecules undergo the same combinatorial chemical reactions, including oxidation in the parent body. Additionally, comparison of chondritic and coal IOM oxidation patterns also did not reveal substantial modifications, which could have taken place during extraction. For example, a use of inert atmosphere prevented oxidation by reactive oxygen under alkali-conditions^43^.

### The range of S-containing compounds in Murchison and Allende IOM

The thermal analysis coupled to analysis of the evolved gases by FTICR MS showed the presence of saturated organosulphur compounds in the Murchison IOM: both CHS and CHOS components. At the same time, XPS analysis detected a lack of these species in the Murchison IOM. Usage of ESI FTICR MS with HDX might be used for specification of the valence state of heteroatoms^28^. We applied that approach to explore the maximum valence state of sulfur in the CHOS species identified in the IOM samples used in our study.

The determination of sulfur-state in the CHOS species is possible by comparison the total number of heteroatoms in the formulae (O, S) and the number of mobile protons, which equals to HDX plus one proton responsible for ionization. At first, the number of mobile protons can be found. And then, the possibility of SO (or -SO_2_, -SO_3_H) can be examined by subtracting from the sum of O + S atoms, which carry mobile protons. For example, the compound with the elemental composition of C_12_H_13_O_5_S_1_ possesses six mobile protons and the same amount of heteroatoms (Fig. 8A): five atoms of oxygen and one sulfur. We suggested that these heteroatoms must belong to five hydroxyl and one thiol groups to provide all six exchanges. A C_13_H_19_O_4_S_1_ compound possesses four mobile hydrogen atoms (Fig. 8B), four oxygen atoms and one sulfur atom. The valence of oxygen atoms in sulfoxide, sulfonyl and sulfonic groups is occupied by bonds with S atoms. For 4 HDX and only 4 O-atoms, in case of C_13_H_19_O_4_S_1_ we can exclude sulfur in oxygenated states. The possible structures may include thiophene or thiol groups, which implies the presence of 4 alcoholic or 1 carboxylic groups, respectively. The presence of the both thiols and thiophenes was also observed by thermogravimetric analysis of IOM.

**Figure 10:**
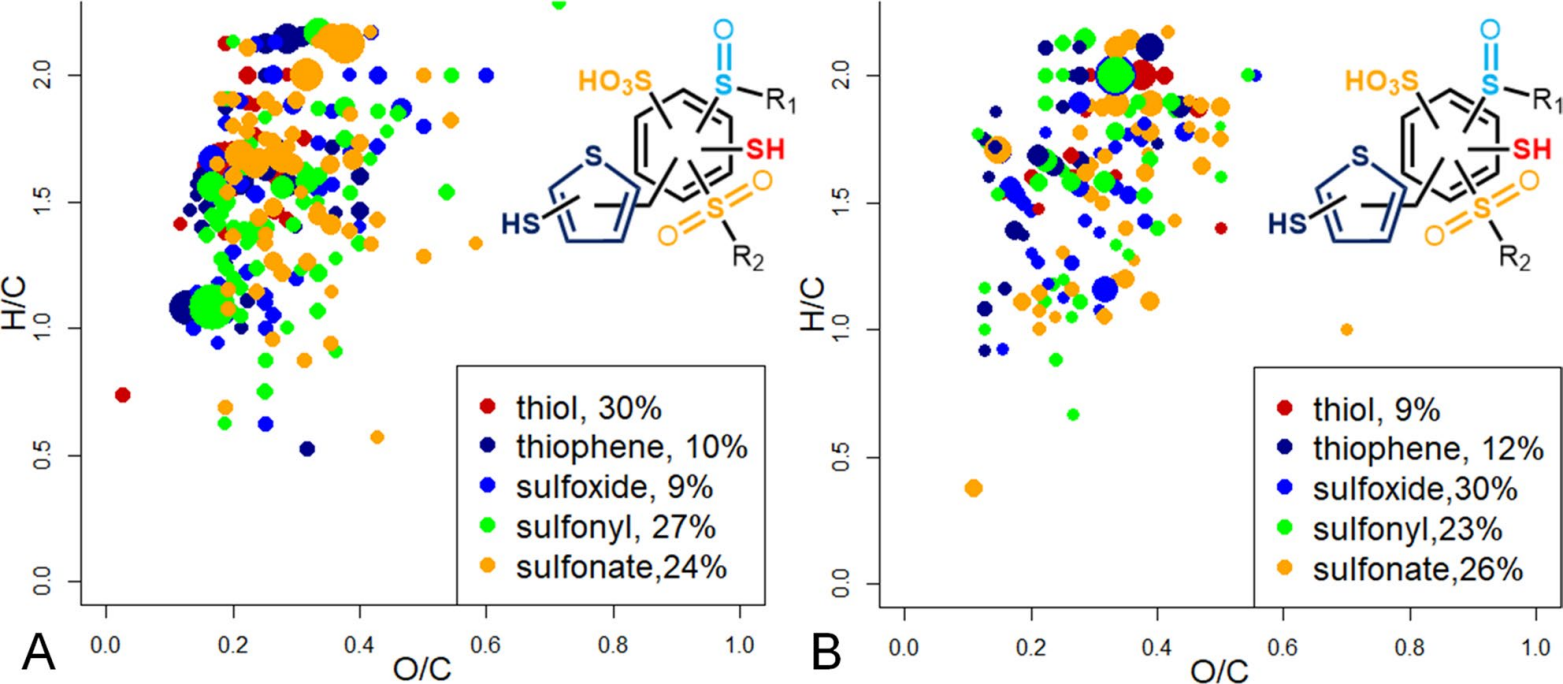
Van Krevelen diagrams with the assigned types of sulfur atoms identified by HDX in alkaline extracts from (**A**) Murchison, (**B**) Allende IOM—thiol, thiol or thiophene, sulfoxide, sulfonyl and sulfonic groups are highlighted in red, dark-blue, blue, green and orange colors, respectfully. The insets show the relative abundance of different sulfur types in CHOS composition.

Following the described algorithm, determination of a valence state of the S-atoms was successfully performed for 199 and 147 CHOS compositions in cases of extracts from Murchison and Allende, respectively. The van Krevelen plots with color-encoded sulfur types are presented in Fig. 10. It was impossible to differentiate between thiol and thiophene groups when the number of mobile protons was smaller than the sum of O + S atoms. As a result, we joined these compartments into one group highlighted in the dark-blue color. Nevertheless, this molecular composition allows us to distinct between aromatic and saturated compounds with H/C > 1.6 using the chemical constraints, which can be put on structure based on a value of the double bond equivalents (DBE) (a sum of multiple bonds and cycles)^37^. For example, the value of DBE for low-oxidized species with H/C > 1.6 is 2 or less, which excludes the presence of conjugated structures like thiophenes, and agrees well with the presence of thiols (highlighted in red in Fig. 10). In addition to the distribution, the relative abundances of S-containing compounds with different sulfur-states were calculated. It was found that the contribution of thiols was significantly higher in case of Murchison as compared to Allende – 30% and 9%, respectively. At the same time, the Allende sample was more abundant with the oxygenated sulfur from sulfoxide to sulfonates, which was in agreement with XPS results. The lower abundance of thiols in the Allende sample is in agreement with its higher petrological type—this sample underwent high temperature alteration, leading to aromatization and oxidation of saturated S-organic compounds^30, 41^.

The results of this study also allowed us to speculate about the nature of both IOM. Allende and Murchison IOM extracts possess 1545 common molecular compositions, which is more than 50% for Allende sample. Application of HDX shows that the samples differ not by the presence of different S-types, but by their contribution. Moreover, the both samples possess thiols, which were previously considered as a result of hydrothermal alteration of IOM^29^. But together our results likely indicate the similar origin of organosulfur compounds (thiols and oxidized S-containing compounds), which were formed at the early stages of accretion. This is supportive of the hypothesis of the accretion of same or similar precursors for IOM formation, which were further exposed to different transformations in the parent bodies^41^.

## Conclusions

Application of FTICR MS to study of the alkali-extracted IOM of Murchison and Allende meteorites revealed their extreme molecular diversity with the significant contribution of relatively oxidized aromatic compounds. A use of alkali extraction allowed us to isolate the portion of IOM whose average characteristics were closer to the bulk IOM composition as compared to the SOM. Unlike previous reports on SOM isolated from CM2 we found the high abundance of CHOS compounds in both Murchison and Allende samples, which occupies wide areas on the corresponding van Krevelen diagrams. We suggested that S-containing compounds in both isolated IOM samples used in this study were comprised of structural fragments with a range of sulfur atom varying in oxidation state from − 2 (in thiols) up to + 6 in (sulfonates). The presence of thiols and the wide range of sulfur states were observed by XPS and thermogravimetry coupled to FTICR MS analyses. The O-based Kendrick mass defect series support the idea of saturated sulfur oxidation. The sulfonates seem to be formed during oxidation of the parent CHOS compounds rather than by a reaction of CHO-species with inorganic sulfates. Applying the combinatorial approach of HDX and FTICR MS we identified the number of protons bonded to heteroatoms for some S-containing ions. A joint assessment of molecular formulae, DBE, KMD and of the HDX results allowed us to determine for the first time the classes of organic compounds for 199 and 147 individual parent-ions of the Murchison and Allende IOM, respectively. For both samples we determined thiols, thiophenes, sulfoxides, sulfonyls and sulfonates. The results of HDX FTICR MS analysis collaborated well with data from XPS and thermogravimetry coupled to FTICR MS analyses. The obtained data and the proposed approach might shed new light on the nature and evolution of the extraterrestrial organic matter.

## Materials and methods

A pieces of the Murchison and Allende meteorites were obtained from the collection of Vernadsky Institute of Geochemistry and Analytical Chemistry of Russian Academy of Science. Both samples were crushed in a planetary mill. The low rank lignite, which was utilized for the comparison to IOM, was obtained from the Senjski coal mine, Serbia. D_2_O (99.9%) was purchased from “Neogaz” company (Russia). The other chemicals were purchased from “Sigma” and were of analytical grade or higher.

### Isolation of the alkali-extractable IOM

IOM fractions of the meteorites were extracted by the modified geochemical approach^20^. A weight of 38 mg of the pulverized Murchison and 35 mg of Allende pieces were suspended three times in CH_2_Cl_2_ to remove terrestrial impurities and lipids followed up by centrifugation and decantation. The solid residues were treated with a mixture of benzene-ethanol (1:2) to extract polar SOM. Demineralization was performed by two consequent treatments with 2 mL of high-purity 6 M HCl and 12 M HF/3 M HCl. Before and after each suspension, solid material was washed with deionized water. Demineralized IOM samples (2.6 mg and 1.3 mg in case of Murchison and Allende, respectively) were dried in vacuum concentrator (Eppendorf) and divided into two parts for further analysis and extraction. Alkali-soluble IOM samples were obtained by modified procedure of humic substances extraction^44^: solid material was soaked in 1 M NaOH and then added with 2 mL of 0.1 M NaOH under Ar atmosphere to prevent oxygenation during extraction. The suspensions were shaken overnight and centrifuged. The solid residues (0.9 mg and 0.5 mg in case of Murchison and Allende, respectively) were dried in vacuum concentrator (Eppendor) for further analysis. The supernatants were acidified and desalted using solid-phase extraction (SPE) on a commercial cartridge (50 mg) filled with modified styrene–divinylbenzene resin (Agilent Bond Elut PPL, Santa Clara, CA, USA) according to the protocol described by Zherebker et al^45^. SPE cartridges were activated by sequential discharge of 1 mL of methanol and 1 mL of 0.01 M HCl. The sample solutions were acidified by 6 M HCl to pH 2 and passed through the activated cartridges at a 4 mL/min flow rate. Then, the cartridges were washed with 6 mL of 0.01 M HCl for salt removal and dried in airflow. The sorbed alkali-soluble fractions of IOM were further eluted by 1 mL of methanol at 1 mL/min flow rate. The collected light-brown eluates were used for FTICR MS measurements. Alkaline extraction of lignite was performed by the same procedure except for demineralization step.

### ESI mass-spectrometric analysis

All experiments were performed on 7 T FTICR MS Apex Ultra (Bruker Daltonics, Germany) equipped with dynamically harmonized cell^46^ and electrospray ionization (ESI) source. For analysis of native samples and performing H/D exchange experiment methanol solutions were diluted two-fold with H_2_O and D_2_O, respectively. The samples were injected into the ESI source using a micro-liter pump at a flow rate of 90 µL/h with a nebulizer gas pressure of 138 kPa and a drying gas pressure of 103 kPa. A source heater temperature of 200 °C was maintained to ensure rapid desolvation of the ionized droplets. The spectra were acquired with a time domain of 4 megawords in ESI (−) and 600 scans were accumulated for each spectrum. Resolving power was 530 000 at *m/z* = 400. Internal calibration was systematically conducted using fatty acids residual signals which yielded accuracy values < 0.5 ppm.

List of peaks with a signal to noise (S/N) ratio exceeding 4 was created using the Bruker Daltonics Data Analysis software. The FTICR MS data were processed using the lab-made software. Charges of ions were determined by calculation of *m/z* distances between parent (^12^C_n_) and isotopic peaks (^13^C_1_^12^C_n-1_). All detected ions were singly charged. Following bulk composition of Murchison IOM—C_100_H_70_O_12_N_3_S_2_^47^ elemental compositions, the generated CHONS formulas were validated by setting sensible chemical constraints (O/C ratio ≤ 1, H/C ratio ≤ 2, element counts (C ≤ 120, H ≤ 200, O ≤ 60, N ≤ 2, S ≤ 1) and mass accuracy window < 0.5 ppm). We did not consider molecular compositions with the higher nitrogen and sulfur content, because of low intensities and S/N ratios of the corresponding peaks in negative ion FTICR mass-spectra. The list of all identified formulae is presented in Appendix A Table S1–3.

The data of labeling experiments were processed using algorithm described in our previous paper^48^. It involves extraction of peaks related to exchange series of individual molecules from the full mass-spectrum by identifying peaks with *m/z* difference of 1.006277 (one H/D exchange). If the parent ion could not be detected in the mass-spectrum of the labeled sample, its position was marked by a red line. Error constraint was set to 0.0005 m*/z*.

### Thermal analysis of Murchison IOM coupled to FTICR MS

For the chemical description of the chemical compounds in Murchison IOM evolved from thermal analysis, a thermobalance (TG 209, Netzsch Gerätebau, Selb, Germany) coupled to a modified Bruker GC-APCI II source, was used^49^. Atmospheric pressure photoionization (APPI) was deployed via a Kr vacuum ultraviolet lamp (10/10.6 eV, 124/117 nm) as a soft ionization technique preserving the molecular pattern and sensitive towards sulphur-containing compounds. 15 mg of the Murchison IOM sample was placed into an aluminum crucible. The temperature program of the thermobalance operated with a constant flow of 200 mL/min nitrogen was 2 min isothermal at 20 °C, ramp to 600 °C with 10 K/min, and hold for 10 min at 600 °C. The high flow rate of inert and high-purity (5.0) nitrogen gas avoids artificial alteration of the sample material. The evolved gas mixture was sampled via a 280 °C interface into the ion source. The mass spectrometric detection was achieved by a Bruker Apex Qe FT-ICR MS equipped with a 7 T superconducting magnet. Mass spectra were recorded summing up ten microscans each from *m/z* 100 to 1000 with a four megaword transient, resulting in a resolving power of roughly 300,000 at *m/z* 400 in positive ion mode. Data analysis and visualization of the temperature resolved thermal analysis mass spectrometric data was carried out using Bruker Data Analysis 5.0 and a self-written Matlab graphical user interface (MATLAB R2018b).

### XPS analysis

The x-ray photoemission spectroscopy (XPS) studies were performed using a PHOIBOS 150 hemispherical analyzer and monochromatic Al Kα radiation with photon energy 1486.61 eV end resolution ∆E = 0.2 eV located at the facility of the Kurchatov complex of synchrotron and neutron investigation (NRC “Kurchatov institute”). The powdered samples were pressed in carbon scotch type and transferred to the vacuum chamber of spectrometer with base pressure 3*10^–9^ mbar. All spectra were measured in fixed analyzer transmission mode with pass energy 120 eV and 40 eV for survey spectra and separated lines, respectively. To analyze the experimental data and decompose the lines into constituent components, the CasaXPS software was used^50^. The NIST laboratory database was used to determine the position of the lines^51^.

## Data availability

Supplementary data and FTICR MS data to this article can be found online at https://www.nature.com/srep/.

## Supplementary Information

Supplementary Information 1.

Supplementary Information 2.
