# Supplementary material for: Speciation of organosulfur compounds in carbonaceous chondrites
Source: Sci Rep. 2021 Apr 1;11:7410. doi: 10.1038/s41598-021-86576-6 (PMC8016918; doi:10.1038/s41598-021-86576-6)
Supplement: Supplementary file 1 — Supplementary Information 1. [file 41598_2021_86576_MOESM1_ESM.docx]

**Supplementary material**

**Speciation of organosulfur compounds in carbonaceous chondrites**

Alexander Zherebker^a^, Yury Kostyukevich^a^, Dmitry S. Volkov^b^, Ratibor G. Chumakov^c^, Lukas Friederici^d^, Christopher P. Rüger^d^, Alexey Kononikhin^a^, Oleg Kharybin^a^, Alexander Korochantsev^e^, Ralf Zimmermann^d,f^, Irina V. Perminova*^b^, Eugene Nikolaev*^a^

^a^ Skolkovo Institute of Science and Technology, 143025 Skolkovo, Moscow region, Russia;

e-mail: [e.nikolaev@skoltech.ru](mailto:e.nikolaev@skoltech.ru)

^b^ Department of Chemistry, Lomonosov Moscow State University, Moscow, Russia. Fax: +7 495 939 5546; e-mail: [iperm@med.chem.msu.ru](mailto:iperm@med.chem.msu.ru)

^с^ National Research Center “Kurchatov Institute”, Moscow, 123182 Russia

^d^ Joint Mass Spectrometry Centre, Chair of Analytical Chemistry, University of Rostock, 18059 Rostock, Germany

^e^ Vernadsky Institute of Geochemistry and Analytical Chemistry of Russian Academу of Sciences, Kosygina 19, Moscow, 119334 Russia

^f^ Joint Mass Spectrometry Centre of Helmholtz Zentrum München, 85764 Neuherberg, Germany

Pages: 4

Pictures: 4

Tables: 3

**Thermogravimetric analysis of Murchison IOM**


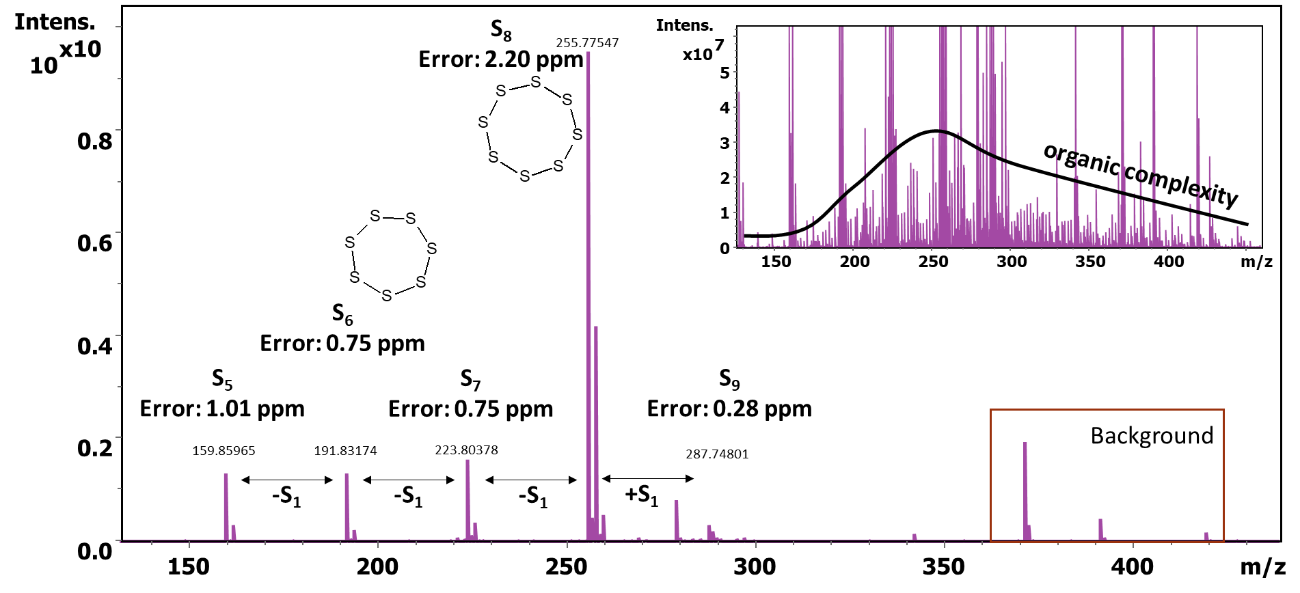


**Figure S1:** Average atmospheric pressure photoionization high-resolution mass spectrum from the thermogravimetric hyphenation (20 °C – 600 °C). The mass spectrometric response is dominated by the decomposition of elemental Sulphur detected as various Sx-compounds. The organic complexity is detected as low abundant distribution from m/z 150 – 450.

**FT ICR MS analysis of alkali extracted coal IOM**

**
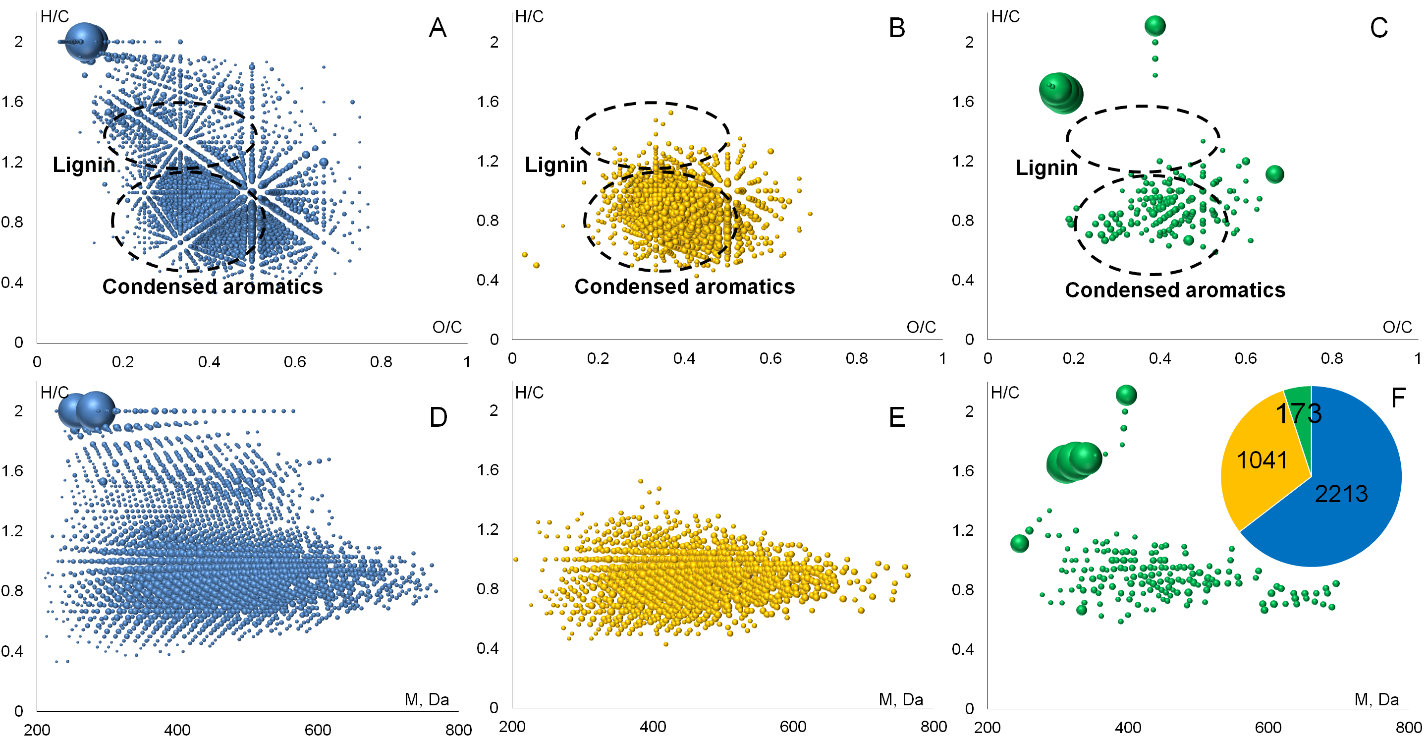
**

**Figure S2.** 3D Van Krevelen (A-C) and H/C versus molecular mass (E-F) diagrams for all molecular compositions identified in the alkali-extracted lignite IOM. The dot size represents relative intensity of the corresponding peak in the FT ICR mass spectrum. CHO, CHON and CHOS compositions are highlighted in blue, orange and green, respectively. Pie chart shows the summarized number of formulae.


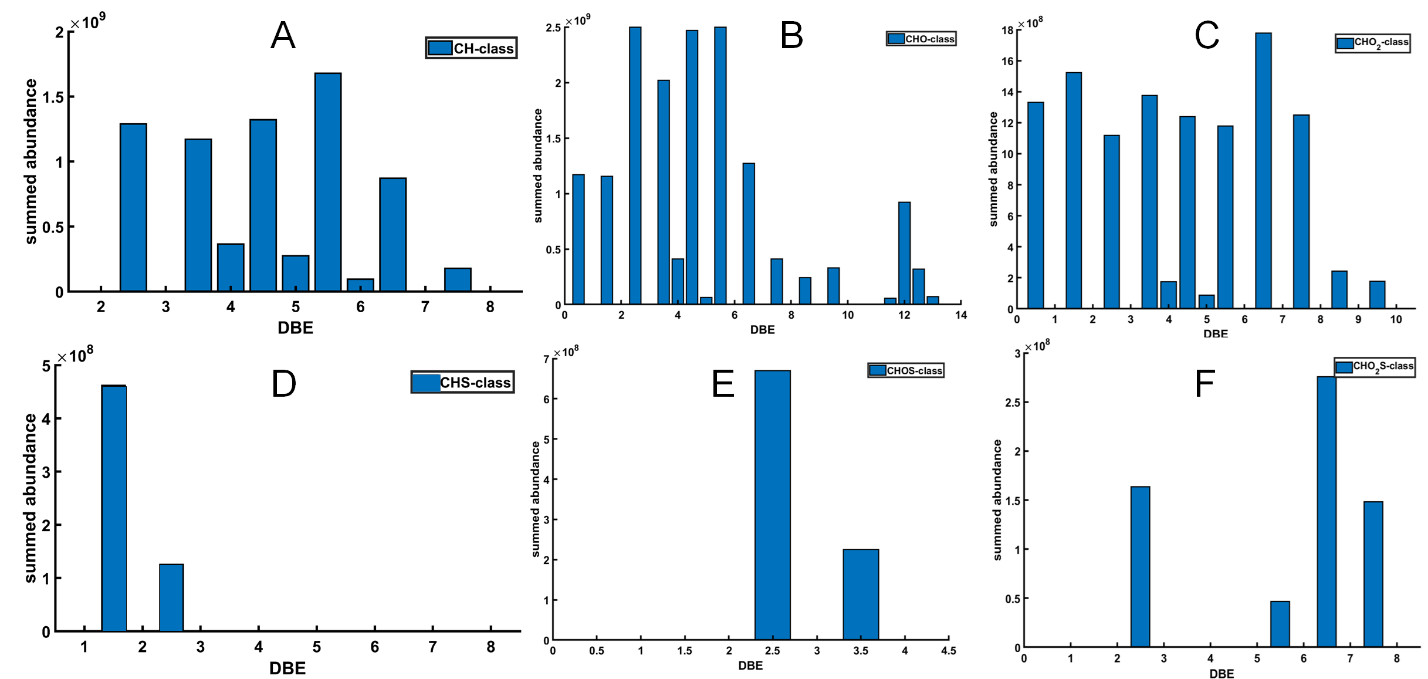


**Figure** S3. DBE distribution of organic classes determined by atmospheric pressure photoionization high-resolution mass spectrum from the thermogravimetric hyphenation in the non-pyrolysis region.


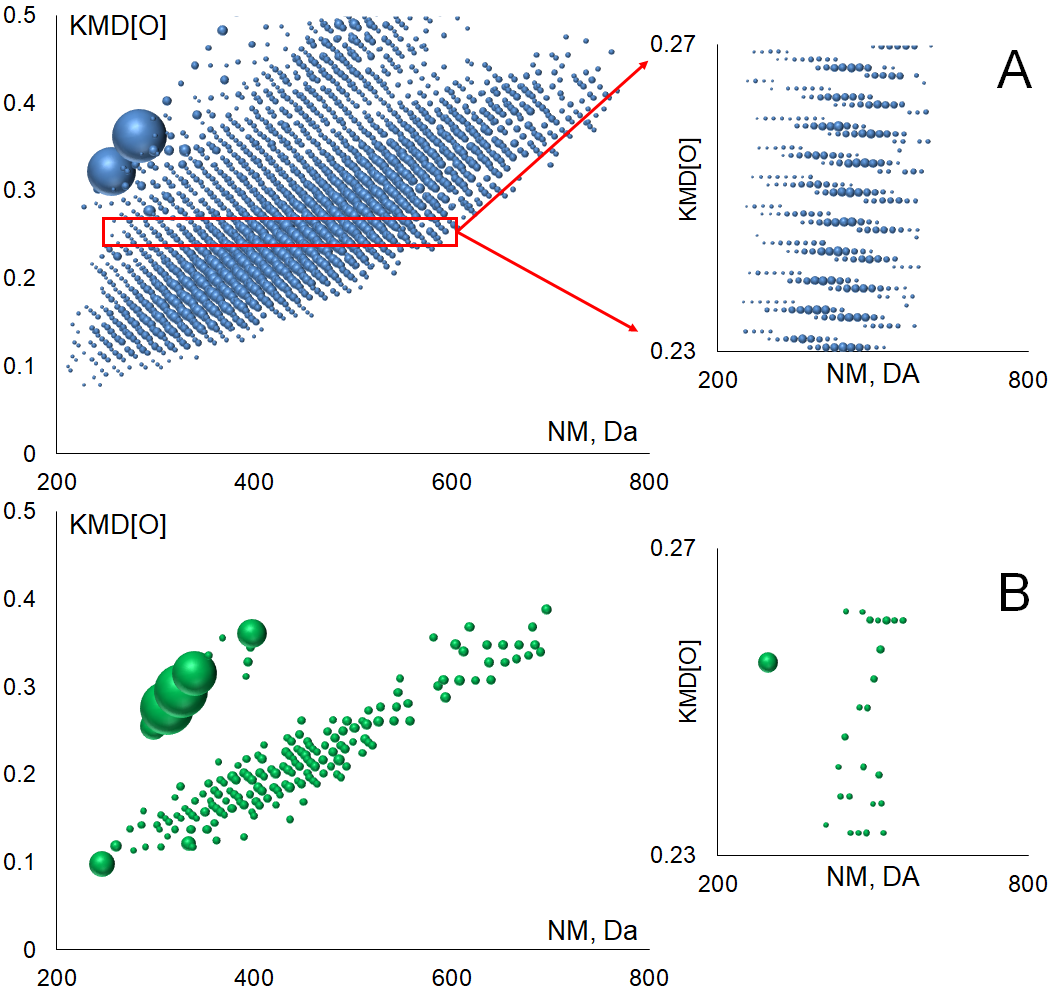


**Figure S4**. O-based Kendrick diagrams for CHO (A, blue) and CHOS (B, green) molecular compositions. Insets show the magnified diagram fragments with several homologues series.

**Table S1**. Composition of identified molecules in alkali-extracted Murchison IOM determined by FT ICR MS.

**Table S2**. Composition of identified molecules in alkali-extracted Allende IOM determined by FT ICR MS.

**Table S3**. Composition of identified molecules in lignite alkali-extracted IOM determined by FT ICR MS.
